# Visualization and Quantification of Transposon Activity in *Caenorhabditis elegans* RNAi Pathway Mutants

**DOI:** 10.1534/g3.119.400639

**Published:** 2019-09-18

**Authors:** Dylan C. Wallis, Dieu An H. Nguyen, Celja J. Uebel, Carolyn M. Phillips

**Affiliations:** Department of Biological Sciences, University of Southern California, Los Angeles, CA 90089

**Keywords:** transposons, double-strand breaks, RNAi, germline, *C . elegans*

## Abstract

RNA silencing pathways play critical roles in maintaining quiescence of transposons in germ cells to promote genome integrity. However the precise mechanism by which different types of transposons are recognized by these pathways is not fully understood. Furthermore, the location in the germline where this transposition occurs after disruption of transposon silencing was previously unknown. Here we utilize the spatial and temporal organization of the *Caenorhabditis elegans* germline to demonstrate that transposition of DNA transposons in RNA silencing pathway mutants occur in all stages of adult germ cells. We further demonstrate that the double-strand breaks generated by transposons can restore homologous recombination in a mutant defective for the generation of meiosis-specific double-strand breaks. Finally, we detected clear differences in transposase expression and transposon excision between distinct branches of the RNA silencing pathway, emphasizing that there are multiple mechanisms by which transposons can be recognized and routed for small-RNA-mediated silencing.

Transposons are discrete segments of DNA that are capable of excising themselves from one locus and reintegrating themselves at another genomic location. Movement of transposons in and out of genes can alter their expression and function, making transposons a major source of deleterious mutations as well as a driving force of evolution. In many organisms, transposons have also been co-opted by researchers for mapping, random and site-directed mutagenesis, and gene tagging (Williams *et al.* 1992; Barrett *et al.* 2004; Williams *et al.* 2005; Robert and Bessereau 2007; Frøkjaer-Jensen *et al.* 2008; Frøkjær-Jensen *et al.* 2010). Because transposons utilize their host’s cellular machinery for their mobilization, they are considered to be selfish DNA parasites, similar to viruses.

There are two major classes of transposable elements – retrotransposons (Class I), which contain an open reading frame coding for a retroviral-like reverse transcriptase and transpose through an RNA intermediate, and DNA transposons (Class II), which move via a DNA-based “cut-and-paste” mechanism. DNA transposons usually contain a transposase sequence flanked by Terminal Inverted Repeats (TIRs). The transposase recognizes the specific sequence of its TIRs and catalyzes a cleavage reaction that releases the transposon ends. The transposase also recognizes a preferred target site, and inserts the transposon at the chosen location (Bessereau 2006). At the site of excision, a DNA transposon leaves behind a double-strand break (DSB), which must be repaired by the host’s cellular machinery, either through homologous recombination or non-homologous end joining. The mechanism of repair is determined primarily based on cell type – somatic cells favor end joining pathways whereas germ cells often repair breaks via homologous recombination, and a subset of these events are resolved as interhomolog crossovers (Plasterk 1991; Robert *et al.* 2008).

There are numerous retrotransposons in the genome, which, until recently, were thought to be inactive. However, a study published in 2012 demonstrated that CER1, Gypsy-like retrotransposon, is transcriptionally active and produces viral-like particles in wild-type *C. elegans* germlines (Dennis *et al.* 2012). More recently, it has been demonstrated that several other retrotransposons, including CER3, are targets of the nuclear RNA interference (RNAi) pathway and H3K9 methylation (Ni *et al.* 2014; 2016; Zeller *et al.* 2016; Ni *et al.* 2018). It is not yet known whether any of these retrotransposons are capable of transposition in *C. elegans*. In contrast, transposition has been detected for at least seven distinct families of DNA transposons (Tc1-Tc5, Tc7, CemaT1), though there are many more transposons present that have not been well studied (Eide and Anderson 1985; Collins *et al.* 1989; Levitt and Emmons 1989; Yuan *et al.* 1991; Collins and Anderson 1994; Rezsohazy *et al.* 1997; Brownlie and Whyard 2004; Bessereau 2006). The most well characterized DNA transposon family in *C. elegans* is Tc1, of which there are 31 intact copies present in the genome (Fischer *et al.* 2003). Tc1 is not normally active in germ cells, however, gene mutations that result in activation of Tc1 were identified from a forward genetic screen and are referred to as *mutator* (*mut*) class genes (Ketting *et al.* 1999). Around the same time, a screen for mutations that result in defects in RNAi identified a largely overlapping panel of genes, suggesting that the silencing of transposons is an endogenous function of the RNAi pathway (Tabara *et al.* 1999).

Many of the *mutator* pathway genes have been identified as components of the small RNA-mediated silencing pathways, including the nucleotidyl transferase (*mut-2/rde-3*), the 3′-5′ exonuclease (*mut-7*), the DEAD box RNA helicase (*mut-14*), the glutamine/asparagine (Q/N)-rich protein (*mut-16/rde-6*), two proteins of unknown function (*mut-8*/*rde-2* and *mut-15*), (Ketting *et al.* 1999; Tijsterman *et al.* 2002; Vastenhouw *et al.* 2003; Tops *et al.* 2005; Chen *et al.* 2005; Gu *et al.* 2009). *C. elegans* with mutations in these genes not only have active transposons and defects in response to exogenous RNAi, but also have temperature-sensitive sterility and defects in endogenous siRNA production (Gu *et al.* 2009; Zhang *et al.* 2011; Phillips *et al.* 2014). All of proteins encoded by these *mutator* pathway genes, along with the RNA-dependent RNA polymerase RRF-1, associate to form a protein complex that synthesizes highly abundant secondary 22G-siRNAs (22 nucleotides starting with a 5′ guanosine) that function downstream of primary Argonaute proteins (Pak and Fire 2007; Sijen *et al.* 2007; Gu *et al.* 2009; Gent *et al.* 2010; Phillips *et al.* 2012). This complex forms nuclear pore-associated perinuclear condensates in germ cells, referred to as *Mutator* foci, where it is thought to play a key role in surveillance and silencing of deleterious transcripts, including transposon-derived RNAs, as they exit the nucleus (Phillips *et al.* 2012; Uebel *et al.* 2018).

In addition to endogenous siRNAs, PIWI-associated small RNAs (piRNAs) also have roles in silencing transposons (Batista *et al.* 2008; Das *et al.* 2008). In *C. elegans*, piRNAs (also referred to as 21U-RNAs) are bound and stabilized by the PIWI protein PRG-1 and trigger production of secondary 22G-siRNAs dependent on the *mutator* pathway (Ruby *et al.* 2006; Wang and Reinke 2008; Batista *et al.* 2008; Das *et al.* 2008; Lee *et al.* 2012; Bagijn *et al.* 2012). Only a single transposon family, Tc3, has been demonstrated to transpose upon loss of the piRNA machinery (Das *et al.* 2008), however, multiple other DNA transposons are up-regulated transcriptionally or lose *mutator* pathway-dependent 22G-siRNAs (Bagijn *et al.* 2012; McMurchy *et al.* 2017).

Here we demonstrate that DSBs generated by transposition of DNA transposons in *mutator* pathway or piRNA pathway mutants can be visualized throughout the germline of adult *C. elegans*, allowing us to determine both temporally and spatially where these transposons are active. Furthermore, in *mutator* pathway mutants these transposon-mediated DSBs can, at some frequency, be repaired by homologous recombination. Thus *mutator* pathway mutants can partially rescue the meiotic defects of *spo-11* mutants, which fail to initiate meiotic recombination through the generation of DSBs. Finally, we observe distinct differences in transposon mRNA expression and frequency of DSBs generated by transposition between *mutator* pathway and piRNA pathway mutants, highlighting the distinct roles these two pathways play in transposon silencing.

## Materials and Methods

### C. elegans strains

Unless otherwise stated, worms were grown at 20° according to standard conditions (Brenner 1974). Strains used in this study include:

N2 – wild-type
AV157 – *spo-11(me44)/nT1 IV; +/nT1 V*
GR1833 – *dpy-3(e27) unc-3(e151) X*
GR1922 – *mut-7(pk204) III; spo-11(me44)/nT1 IV; +/nT1 V*
GR1923 – *mut-16(pk710) I; spo-11(me44)/nT1 IV; +/nT1 V*
NL1810 – *mut-16(pk710) I*
PD4792 – *mIs11 IV*
SX922 – *prg-1(n4357) I*
USC222 – *mIs11 IV; dpy-3(e27) unc-3(e151) X*
USC223 – *mut-16(pk710) I; mIs11 IV; dpy-3(e27) unc-3(e151) X*
USC313 – *prg-1(n4357) I; spo-11(me44)/nT1 IV; +/nT1 V*

### RNA isolation and quantitative RT-PCR

RNA was isolated from synchronized adult *C. elegans* (66-68 h after L1 arrest) using Trizol, followed by chloroform extraction and isopropanol precipitation. qRT-PCR was performed using transposon-specific primer pairs and *rpl-32* for normalization (Table 1). Data were analyzed using the 2^-ΔΔCt^ method and P-values were calculated in R using the *t*-test function in the package ‘pcr’ (Ahmed and Kim 2018).

**Table 1:** Primers used in this study

Name	Sequence
Tc1 - F	TGGGCTAAACACATCTGGTC
Tc1 - R	CGGTTGGGCATTGATACTTTG
Tc2 - F	AGTTATGAGGATTGGATGGTGC
Tc2 - R	AGTATTGGAGCATTGACGGC
Tc3 - F	GTCCGTATCGTGTATGCTCAG
Tc3 - R	AATAGACTTCCAAGCGTCGAG
Tc4v - F	GTAATCGCTGAACCAAAAGGC
Tc4v - R	GTGTCTTGTATCCAGCCCG
Tc5 - F	AGTGTACCGTGTCTTTCGTG
Tc5 - R	GGAGTTTCCACTTTGACATGTTG
RTE1 - F	CCCTGGAATGAGAGTGAATGG
RTE1 - R	GTACGAGTTCTTGGAGCATTTTG
CER1/Gypsy - F	CCCGGAACTATGCTCATTCTAG
CER1/Gypsy - R	TCAGTACAGACGAAGCAGTTC
Mirage - F	AGAAGCTGAAACCGATGAGTC
Mirage - R	TCAGAGAACGACACAGTTGAC
*rpl-32* - F	CAAGGTCGTCAAGAAGAAGC
*rpl-32* - R	GGCTACACGACGGTATCTGT

### Fluorescent microscopy

*C. elegans* were picked as L4s and dissected the following day for immunofluorescence for most experiments. For diakinesis imaging and scoring, animals were picked as L4 and kept for three days at 15**°** prior to dissection. All strains carrying the *spo-11* mutation were selected as L4s from the progeny of balanced *spo-11/nT1* animals. Gonads were immunostained according to previously described protocol with rabbit anti-RAD-51 (SDIX, 2948.00.02), guinea pig anti-HTP-3 (MacQueen *et al.* 2005), and Alexa Fluor secondary antibodies purchased from ThermoFisher (Phillips *et al.* 2009). Imaging was performed on an Axio Imager Z1 microscope with ApoTome running Axiovision software (Zeiss) or a DeltaVision Elite microscope running SoftWoRx (GE Healthcare). Images were collected as three-dimensional data stacks, displayed as maximum intensity projections, and pseudocolored using Adobe Photoshop.

### RAD-51 quantification

Age-matched (one day post-L4) hermaphrodite gonads were immunostained for RAD-51 and imaged. Gonads were divided into six zones of equal length, starting at the distal tip through the end of pachytene and the number of foci per nucleus were scored for each zone. Three gonads were scored for each genotype.

### Brood size analysis

Hermaphrodites of the indicated genotypes were placed on individual plates as L4-stage larvae. They were moved to fresh plates approximately every 24 hr until egg laying was complete. At the time the animal was removed from the plate, the total number of embryos and hatched larvae was counted. Approximately three days later, the total number of hermaphrodite and male progeny on the plate was scored. Total number of broods scored was 11 broods for wild-type, 29 broods for *spo-11*, 13 broods for *mut-7*; *spo-11*, 15 broods for *mut-16*; *spo-11*, and 21 broods for *prg-1*; *spo-11*.

### Recombination analysis

Hermaphrodites heterozygous for *dpy-3unc-3* and homozygous for *spo-11* were generated by mating balanced *spo-11/**mIs11* males to *mIs11*; *dpy-3unc-3* hermaphrodites. The transgene *mIs11* is located on chromosome IV near the *spo-11* locus; it can be identified by a pharyngeal GFP signal and was used to balance the *spo-11* mutant. The *spo-11*/*mIs11*; *dpy-3unc-3* F2 hermaphrodites were then mated to *spo-11*/*mIs11* males to generate the *spo-11*; *dpy-3unc-3*/++** strain used for recombination analysis. The cross was performed similarly using *mut-16/+*; *spo-11/**mIs11* males and *mut-16*; *mIs11*; *dpy-3unc-3* hermaphrodites to generate the *mut-16*; *spo-11*; *dpy-3unc-3/++* and the *mut-16/+*; *spo-11*; *dpy-3unc-3/++* strains, which were identified as homozygous or heterozygous for *mut-16* by genotyping after egg-laying was completed. Progeny from each cross were scored as wild-type, Unc Dpy, Unc non-Dpy, or Dpy non-Unc, and as hermaphrodite or male. Recombination frequency (*p*) for hermaphrodites was calculated as *P* = 1 – $\sqrt{\left(1-2R\right)}$ , where *R* is the fraction of recombinant progeny, scored as two times the number of Unc non-Dpy hermaphrodites to account for the possibility that Dpy non-Unc animals are non-recombinant triplo-X animals (Brenner 1974). For males, recombination frequency is calculated as *P* = *R*. The total recombination frequency is calculated ((2 * *p*_hermaphrodite_ * [# of hermaphrodites]) + (*p*_male_ * [# of males]))/(2 * [# of hermaphrodites] + [# of males]), which accounts for hermaphrodites having two X chromosomes and males only one (Kelly *et al.* 2000). Map distances in cM = 100 × *p*. Total number of broods scored was 28 broods for *spo-11*; *dpy-3 unc-3/++*, 7 broods for *mut-16/+*; *spo-11*; *dpy-3 unc-3/++*, and 9 broods for *mut-16*; *spo-11*; *dpy-3 unc-3/++*.

### Data Availability

All strains are available either at the Caenorhabditis Genetics Center (CGC) or upon request from the Phillips lab. The authors affirm that all data necessary for confirming the conclusions of the article are present within the article, figures, and tables.

## Results

### Transposon mRNA expression profiles of RNAi pathway mutants

**Figure 1:**
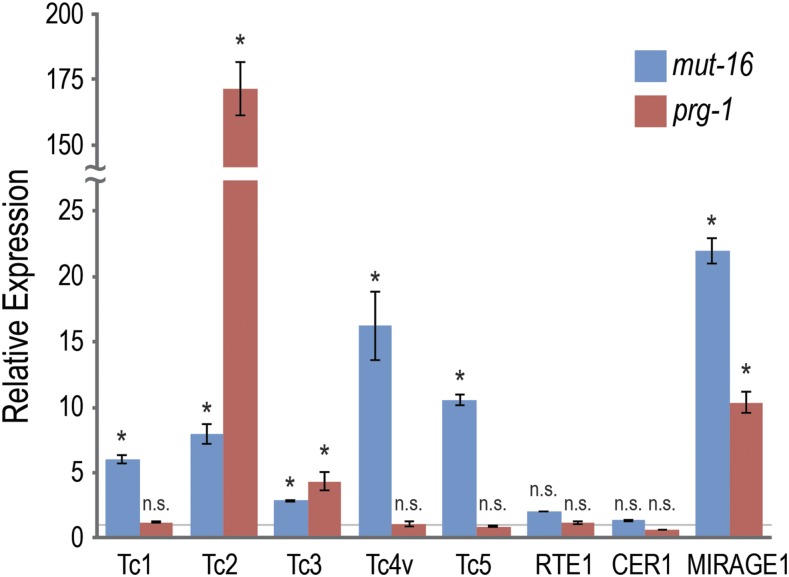
*Mutator* pathway mutants and piRNA pathway mutants have distinct profiles of transposon mRNA expression. Primers recognizing the transposon mRNAs were used for quantitative RT-PCR with *rpl-32* as a normalization standard. Expression levels shown are relative to wild-type animals (gray horizontal line) and error bars represent the standard deviation of two technical replicates. Two primer sets were used for each transposon mRNA with similar results, however only one representative set is shown. n.s denotes not significant and indicates p-value > 0.05 and * indicates p-value < 0.05.

It is well known that RNAi pathways regulate DNA transposon activity in the *C. elegans* germline (Billi *et al.* 2014). However, the specific temporal and spatial region of the germline where this transposition occurs has not previously been studied. To address this question, we sought to visualize transposon activity by utilizing the DSBs left behind by the DNA transposons when they transpose, which are then repaired by cellular DNA repair machinery. First, however, we sought to determine how transposon activity differs between distinct branches of the RNAi pathway. It was previously reported that mutants in the *mutator* pathway and the piRNA pathway have different rates of transposon mobilization depending on the transposon being examined. For example, Tc1, Tc3, and Tc4 transposons are active in a *mut-7* mutant, whereas only the Tc3 transposon is active in a *prg-1* mutant (Das *et al.* 2008). As a preliminary analysis of which transposons may mobilize in mutants from either the *mutator* pathway or the piRNA pathway, we performed qRT-PCR analysis of transposon mRNA expression in *mut-16* and *prg-1* mutants (Figure 1). Specifically, we examined the mRNA expression from several known DNA transposons (Tc1, Tc2, Tc3, Tc4v, Tc5, and MIRAGE1) and two retrotransposons (RTE1 and CER1/Gypsy) and found that, of the DNA transposons, Tc1, Tc4v and Tc5 had significantly increased mRNA expression in *mut-16* but not *prg-1* mutants, whereas Tc2, Tc3, and MIRAGE1 had increased expression in both mutants. Interestingly, Tc2 was significantly higher in *prg-1* (170-fold) compared to *mut-16* (eightfold), which is surprising because piRNA-mediated silencing is generally thought to be upstream of and to require the *mutator* pathway (Das *et al.* 2008; Lee *et al.* 2012; Bagijn *et al.* 2012). It is important to note that this analysis is only indicative of transposon mRNA expression in the RNAi pathway mutants relative to wild-type animals, and is not direct evidence of transposon mobilization rates. Furthermore, while this analysis does not distinguish between somatic and germline transposon activity, it does suggest that Tc2 transposon silencing may be mediated, at least in part, by a piRNA pathway that is independent of the *mutator* pathway and WAGO 22G-siRNAs.

### Visualization and quantification of transposon mobilization

**Figure 2:**
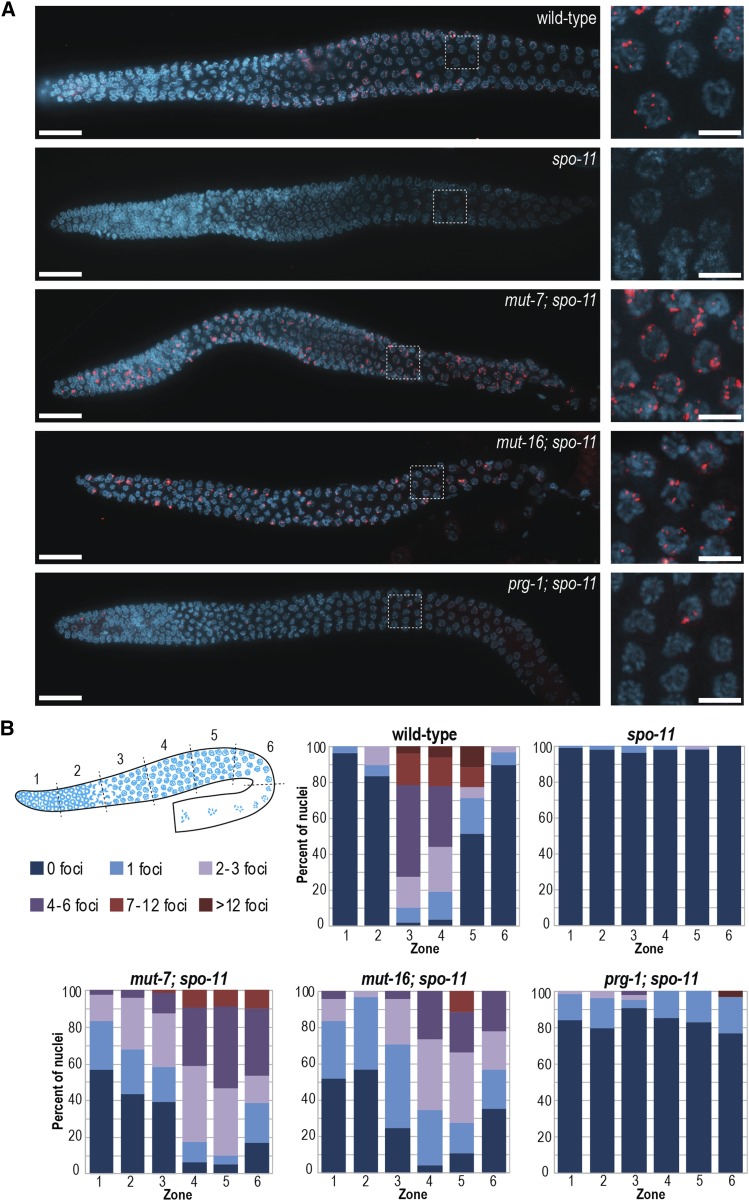
SPO-11-independent RAD-51 foci are present throughout the germlines of *mutator* pathway mutants. (A) Whole gonads (left) stained with RAD-51 (red) and DAPI (blue). RAD-51 foci can be seen throughout the germlines of *mut-7*; *spo-11*, *mut-16*; *spo-11*, and to a lesser extent, *prg-1*; *spo-11*. This is in contrast to wild-type, where the majority of RAD-51 foci are found in the zygotene to mid-pachytene region. Scale bars, 20μm. Magnification of pachytene stage nuclei (right) stained with RAD-51 (red) and DAPI (blue). Scale bars, 5μm. (B) Diagram (top left) depicting the six zones in which RAD-51 foci were quantified. Stacked bar charts show percent of nuclei in each zone with the specified number of RAD-51 foci for the indicated genotypes. X axes indicate the position in the germline (zone).

To visualize transposon activity specifically in the *C. elegans* germline, we chose to examine the expression of RAD-51, a homolog of the bacterial RecA protein and a key protein in DSB repair pathways (Ogawa *et al.* 1993). In wild-type *C. elegans*, RAD-51 can be visualized as distinct, punctate foci in the zygotene/pachytene stages of meiosis (Figure 2A) (Alpi *et al.* 2003). We initially examined multiple mutants in the RNAi pathway for increased RAD-51 foci in germ cells, however, the presence of programmed DSBs generated for meiotic recombination, complicated the analysis. To alleviate this problem, we crossed the RNAi pathway mutants into a *spo-11* mutant. SPO-11 is the type-II topoisomerase that is required to initiate meiotic recombination through the generation of DSBs (Keeney *et al.* 1997; Dernburg *et al.* 1998). In the *spo-11* mutant, RAD-51 foci are virtually eliminated (Figure 2A-B) (Alpi *et al.* 2003), providing us a background where we can examine *spo-11*-independent DSBs generated due to transposon mobilization. We first examined the germline of *mutator* mutants (*mut-7* or *mut-16*) in the *spo-11* background. In these strains we could visualize numerous DSBs throughout the germline, starting in the mitotic proliferation zone, and extending through the meiotic stages of leptotene, zygotene, pachytene, and diplotene (Figure 2A-B). Because the number of foci increases as the nuclei progress through the meiotic program in the *mutator* pathway mutants, we can infer that transposons are generating new DSBs throughout these stages. Additionally, we examined the germline of *C. elegans* with a mutation in the piRNA pathway (*prg-1*). In *prg-1* mutants we observed significantly fewer DSBs compared to the *mutator* mutants, but higher levels than in the *spo-11* mutant alone (Figure 2A-B). These results are consistent with a role for the piRNA pathway in silencing only a subset of transposons, in contrast to the *mutator* pathway, which is more broadly required for transposon silencing.

### Transposon-induced DSBs can partially rescue meiotic defects of spo-11 mutants

We next sought to examine whether transposon-induced DSBs are competent to rescue the meiotic phenotypes of the *spo-11* mutant. In the absence of functional SPO-11 protein, chromosomes fail to undergo meiotic recombination (Keeney *et al.* 1997; Dernburg *et al.* 1998). Failure to undergo meiotic recombination causes errors in segregation of chromosomes at the meiosis I division, ultimately resulting in aneuploidy and embryonic lethality. The few progeny surviving to adulthood from *spo-11* mutants are frequently males (Dernburg *et al.* 1998). This “High incidence of males” or Him phenotype is also indicative of a chromosome segregation defect; male *C. elegans* have a single sex chromosome (XO) and thus mis-segregation of the X chromosomes in an XX hermaphrodite results in an increased production of males (Hodgkin *et al.* 1979). In contrast to the *spo-11* null mutants, which largely produce inviable embryos that fail to survive to adulthood (2.8% viable), *mut-7*; *spo-11* and *mut-16*; *spo-11* produce 12.1% and 10.7% viable embryos that survive to adulthood, respectively (Table 2). Similarly, *mut-7*; *spo-11* and *mut-16*; *spo-11* produce fewer male progeny (14.9% males for *mut-7*; *spo-11* and 21.4% males for *mut-16*; *spo-11*) than the *spo-11* mutant alone (40.9% males) (Table 2). Unlike the *mutator* pathway mutants, the piRNA pathway mutant *prg-1* failed to rescue embryonic viability or the production of male progeny. These data indicate that the DSBs generated by transposon mobilization in the *mutator* pathway mutants, but not piRNA pathway mutants, can compensate for the lack of SPO-11 protein and increase the frequency of proper chromosome segregation, presumably by promoting the formation of crossovers.

**Table 2:** *Mutator* pathway mutations increase progeny viability and reduce the number of self-progeny males in a *spo-11* mutant

Genotype	% Viable Embryos*^a^*	% Male Progeny*^b^*
wild-type	100.00 (n = 3035)	0.07 (n = 3035)
*spo-11*	2.80 (n = 5328)	40.94 (n = 149)
*mut-7*; *spo-11*	12.11 (n = 1882)	14.91 (n = 228)
*mut-16*; *spo-11*	10.68 (n = 2144)	21.40 (n = 229)
*prg-1*; *spo-11*	2.12 (n = 4105)	51.72 (n = 87)

a Total number of embryos scored to calculate % viable embryos indicated in parentheses.

b Total number of adults scored to calculate % male self-progeny indicated in parentheses.

**Figure 3:**
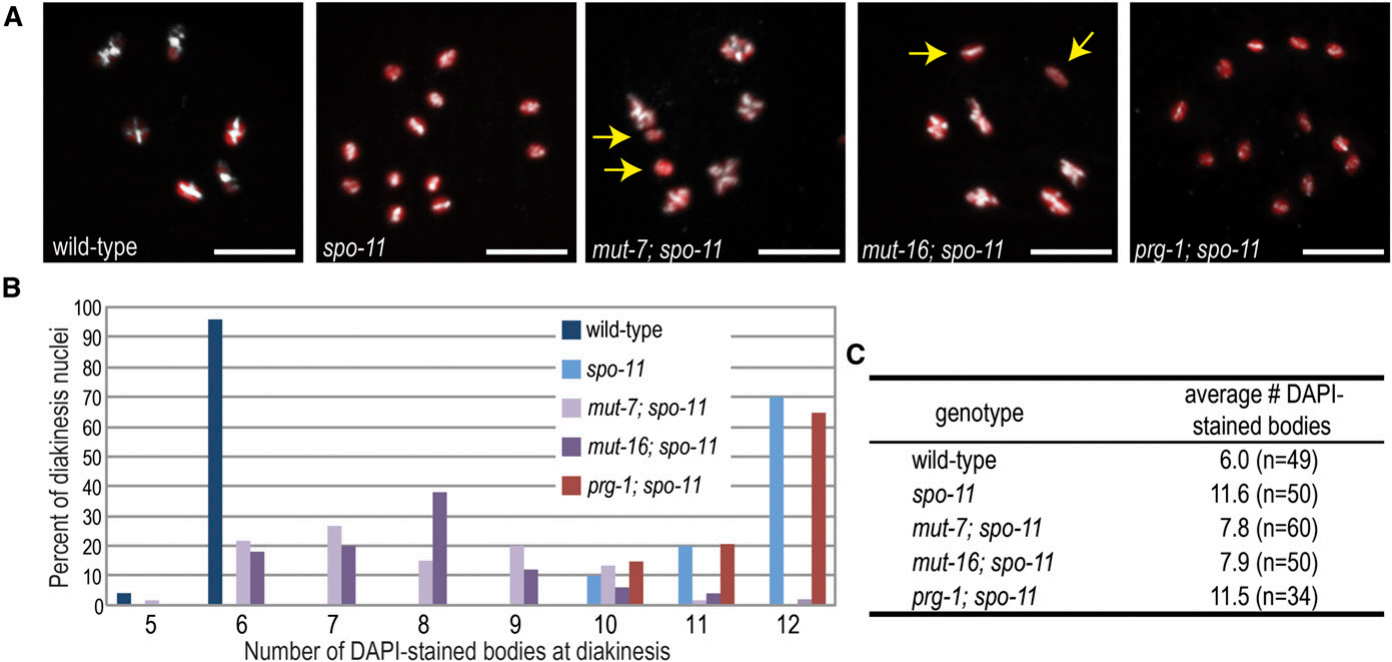
Mutations in the *mutator* pathway can restore crossover formation in the *spo-11* mutant. (A) Representative wild-type and mutant diakinesis oocytes stained with HTP-3 (white) and DAPI (red) to allow for counting of the number of the number of bivalents (homologous chromosomes connected by chiasmata) or univalents in each strain. Yellow arrows in *mut-7*; *spo-11* and *mut-16*; *spo-11* point to a single pair of non-recombinant chromosomes. Scale bars, 5μm. (B) Graph indicating the number of DAPI-stained bodies in diakinesis oocytes for each genotype. Wild-type oocytes display six DAPI-stained bivalents, representing the six pairs of chromosomes held together by chiasmata, while *spo-11*, which fails to make double-strand breaks for recombination, displays 12 DAPI-stained univalents. Mutations in the *mutator* pathway but not the piRNA pathway can partially rescue the *spo-11* phenotype. Occasionally, two bivalents lie too close together to be visually resolved, resulting in a modest underestimation of the number of DAPI-stained bodies. (C) Mean number of DAPI-stained bodies scored for each of the genotypes in (B). Total number of oocytes scored is indicated in parentheses.

To test directly whether DSBs generated by transposon mobilization can promote the formation of crossovers, we examined diakinesis stage of meiosis for the presence of recombinant chromosomes. In wild-type *C. elegans*, six bivalents (pairs of recombinant chromosomes) are present, whereas, in *spo-11*, 12 non-recombinant univalents can be observed (Villeneuve 1994; Dernburg *et al.* 1998). The *mutator* pathway mutants, *mut-7* and *mut-16*, were able to partially rescue the *spo-11* diakinesis phenotype, averaging approximately eight DAPI-staining bodies, but with a range of six to 11 DAPI-staining bodies (Figure 3A-C). In contrast, the piRNA pathway mutant, *prg-1*, was indistinguishable from the *spo-11* mutant alone with 12 univalents (Figure 3A-C). We also analyzed the frequency of recombination between two genetic markers, *dpy-3* and *unc-3*, which lie on opposite ends of the X chromosome, a distance of ∼38 cM in wild-type animals (Villeneuve 1994; Dernburg *et al.* 1998; Kelly *et al.* 2000). In *spo-11* mutants, recombination is undetectable in this region (Dernburg *et al.* 1998) whereas *mut-16*; *spo-11* mutants we calculated the map distance between *dpy-3* and *unc-3* as 26.6 cM (∼70% of wild-type) (Table 3). These data indicate that, in each germline nucleus of a *mutator* pathway mutant, most chromosomes have at least one mobilized transposon generating a DSB that is subsequently repaired by homologous recombination. Because some DSBs are occurring well before or after the stage at which nuclei are competent for homologous recombination and because many DSBs may be repaired by other mechanisms, these figures significantly underestimate the total number of mobilized transposons per nucleus.

**Table 3:** A mutation in the *mutator* pathway restores recombination in the *spo-11* mutant

	Recombinant hermaphrodites			Recombinant males			
Genotype	Unc non-Dpy hermaphrodites	Dpy non-Unc hermaphrodites	Total hermaphrodites	Unc non-Dpy males	Dpy non-Unc males	Total males	Map distance (cM)*^a^*
*+/+* or *mut-16/+*; *spo-11*; *dpy-3 unc-3/++*	0	0	134	0	0	131	0.0
*mut-16*; *spo-11; dpy-3 unc-3/++*	27	29	239	9	10	60	26.6

a Map distance was calculated as described in Materials and Methods.

## Discussion

By visualizing transposon-derived DSBs as RAD-51 foci present in a *spo-11* mutant background, we can provide quantification of transposon-hopping levels in the *mutator* pathway and piRNA pathway mutant backgrounds. Furthermore, in *mutator* mutants, transposon-derived DSBs can rescue *spo-11* mutant phenotypes, including recombination frequency, chiasma formation, viability, and male production. The assays described could be extended to examine new mutants in the transposon-silencing pathway, to screen for new mutants by taking advantage of the increased fertility of *spo-11* mutants when combined with mutations in the transposon silencing pathway, or to probe more deeply into which classes of transposons are mobilized and the frequency using ChIP-seq of RAD-51.

Interestingly, we observe clear differences in the expression of transposon mRNAs by qRT-PCR and in the rates of transposition assayed by frequency of DSBs, demonstrating that these two pathways do not have fully overlapping roles in transposon silencing. This result, along with previously reported differences in Tc1 and Tc4 mobilization between the two pathways (Das *et al.* 2008), is somewhat surprising because piRNA pathways are thought to be the primary mediator of transposon silencing in many organisms (Czech and Hannon 2016). That leads to the question of how transposons silenced independently of piRNAs are recognized. piRNA-targeting can trigger multigenerational silencing that can be maintained in the absence of the initial piRNA trigger (Ashe *et al.* 2012; Shirayama *et al.* 2012; Luteijn *et al.* 2012). Thus one possibility is that silencing of these transposons was initiated by piRNAs, but when those piRNAs were lost, silencing was maintained by *mutator*-dependent heritable siRNAs. In fact, a mutation in *hrde-1*, the Argonaute protein required to inherit siRNAs from one generation to the next, in combination with a mutation in *prg-1*, desilences the Tc1 transposon to a level similar to that of a mutation in the *mutator* pathway (de Albuquerque *et al.* 2015). Alternatively, some features of the transposon mRNA structure could be recognized by the cell as aberrant and routed for silencing completely independently of piRNAs. For example, multiple reports have implicated splicing factors in the RNA silencing pathway, suggesting that irregular introns or mis-spliced mRNAs can be a signal for siRNA-mediated silencing (Kim *et al.* 2005; Dumesic *et al.* 2013; Akay *et al.* 2017; Tyc *et al.* 2017; Newman *et al.* 2018). Many *C. elegans* transposons contain introns, including Tc1 and Tc3 (van Luenen *et al.* 1993; Vos *et al.* 1993). Interestingly, the Tc1 intron is inefficiently spliced, yet 22G-siRNAs are exclusively made from spliced transcripts which accumulate in spliceosomes, suggesting that siRNA biogenesis is downstream of splicing-mediated surveillance (Sijen and Plasterk 2003; Newman *et al.* 2018). How these surveillance mechanisms are interwoven to mediate efficient recognition of transposon and other foreign mRNAs remains to be determined.
